# A Machine Learning Model for Accurate Prediction of Sepsis in ICU Patients

**DOI:** 10.3389/fpubh.2021.754348

**Published:** 2021-10-15

**Authors:** Dong Wang, Jinbo Li, Yali Sun, Xianfei Ding, Xiaojuan Zhang, Shaohua Liu, Bing Han, Haixu Wang, Xiaoguang Duan, Tongwen Sun

**Affiliations:** ^1^General Intensive Care Unit, The First Affiliated Hospital of Zhengzhou University, Zhengzhou, China; ^2^Key Laboratory for Critical Care Medicine of Henan Province, Zhengzhou, China; ^3^Key Laboratory for Sepsis of Zhengzhou, Zhengzhou, China; ^4^Department of Electrical and Computer Engineering, University of Alberta, Edmonton, AB, Canada

**Keywords:** sepsis, machine learning, prognostication, infection, ICU patients

## Abstract

**Background:** Although numerous studies are conducted every year on how to reduce the fatality rate associated with sepsis, it is still a major challenge faced by patients, clinicians, and medical systems worldwide. Early identification and prediction of patients at risk of sepsis and adverse outcomes associated with sepsis are critical. We aimed to develop an artificial intelligence algorithm that can predict sepsis early.

**Methods:** This was a secondary analysis of an observational cohort study from the Intensive Care Unit of the First Affiliated Hospital of Zhengzhou University. A total of 4,449 infected patients were randomly assigned to the development and validation data set at a ratio of 4:1. After extracting electronic medical record data, a set of 55 features (variables) was calculated and passed to the random forest algorithm to predict the onset of sepsis.

**Results:** The pre-procedure clinical variables were used to build a prediction model from the training data set using the random forest machine learning method; a 5-fold cross-validation was used to evaluate the prediction accuracy of the model. Finally, we tested the model using the validation data set. The area obtained by the model under the receiver operating characteristic (ROC) curve (AUC) was 0.91, the sensitivity was 87%, and the specificity was 89%.

**Conclusions:** This newly established machine learning-based model has shown good predictive ability in Chinese sepsis patients. External validation studies are necessary to confirm the universality of our method in the population and treatment practice.

## Introduction

Although numerous studies and papers on sepsis are published every year, it remains a major challenge for patients and clinicians worldwide. Between 2002 and 2012, the proportion of sepsis patients admitted to hospitals in the European ICU remained unchanged; however, the severity of the disease increased significantly (1). The standardized sepsis-related mortality rate in China in 2015 was 67 deaths per 100,000, which was equivalent to more than 1 million deaths due to sepsis (2). Despite these alarming numbers, the public seems to lack an awareness about sepsis. An adult survey found that <30% of people are aware of the severity of sepsis, which was much lower than the proportion for cardiovascular diseases, cancer, and asthma (3).

To date, the diagnosis of sepsis has largely relied on determining the presence of infection and organ dysfunction (4). In addition, screening laboratory tests are often required to confirm the diagnosis. However, laboratory testing takes time, so treatment is further delayed (5).

The early detection and prediction of patients who may develop sepsis is essential to improve the adverse consequences of sepsis. Although there are many studies on the early predictions of sepsis, such as calcitonin, C-reactive protein, white blood cells, platelets, and lactic acid (6, 7). However, disappointingly, most studies are limited in clinical prediction (8). Since sepsis is a complex clinical syndrome, it contains a wide range of multifaceted clinical and biological features; therefore, a single clinical index may not be a good reflection of the disease state (9). There is still a lack of effective biomarker combinations that can distinguish patients with sepsis from those not affected with sepsis.

Current research mainly uses data collected by bedside monitors to determine the probability of sepsis in ICU patients. Bloch et al. constructed a sepsis prediction model based on the four vital signs of mean arterial pressure, heart rate, respiratory rate, and body temperature (10). The best area under the curve (AUC) was achieved with Support Vector Machine (SVM) with radial basis function, which was 88.38%. Guillén et al. used vital sign measurements and laboratory test results to predict the likelihood of severe sepsis in patients with sepsis during ICU hospitalization (11). The study showed that the AUROC based on vital signs data was 0.84; based on vital signs and laboratory results, the AUROC was calculated to be 0.882. Calvert et al. studied the correlation between pairs and triples of vital sign measurements and the overall trend (i.e., increase, decrease, and no change) of the measurements over time to predict sepsis in the adult ICU population disease (12). Their results show that the average AUROC measurement accuracy is 0.83, but requires a larger data set, which usually requires longer processing time. Since the above studies are based on the previous definition of the Third International Consensus Definition of Sepsis (sepsis-3), our current understanding of sepsis is of limited reference value. Nemati et al. used electronic medical record data combined with high-resolution time series of heart rate and blood pressure to dynamically predict sepsis, with an area under the receiver operating characteristic (AUROC) of 0.83–0.85 (13). Although the study is based on the third international definition of sepsis (sepsis-3), its predictive power is not significantly different from previous studies.

Machine learning has been applied to multiple healthcare fields, including diabetes, cancer, cardiology, and mental health (14–17). Most of the machine learning models and tools developed in the research environment has studied the potential of prognosis, diagnosis, or clinical componentization, thus demonstrating the prospect of developing computerized decision support tools (18, 19). In general, the use of machine learning models can improve patient safety, improve the quality of care, and reduce medical costs (20).

The application of artificial intelligence in the medical field is gaining increasing recognition in the improvement of clinical practice and achievement of personalized treatment (21, 22). This study used machine learning methods to evaluate predictive clinical indicators and biomarkers related to sepsis and to establish a model that could effectively predict sepsis early, which is necessary to identify high-risk patients and may enhance the understanding and facilitate clinical management of sepsis.

## Materials and Methods

### Study Population

This study was a secondary analysis of a retrospective observational study conducted from 2014 to 2016 in the intensive care unit (ICU) of the First Affiliated Hospital of Zhengzhou University. The inclusion criteria were (1) infection at the time of admission to the ICU; (2) compliance with the international consensus definition of sepsis and septic shock (Sepsis-3.0); (3) age ≥18 years. The exclusion criteria were (1) age <18 years; (2) diseases without infection status such as coronary heart disease, cardiac arrest, fracture, neoplasm, cerebral infarction, and brain injury; and (3) more than three missing data. Clinical or laboratory parameters related to infection and sepsis were collected for each patient.

### Statistical Analysis

The binary variables were described as counts and percentages and were evaluated using the Chi-square test or Fisher's exact test. If the continuous variables conformed to a normal distribution, they were compared using a *t*-test and expressed as means ± SEM. For a non-normal distribution, the Mann–Whitney *U* test was used. *P* < 0.05 was considered statistically significant. The ensemble model was written Python scripting language (Version 3.6.5, Python Software Foundation, Wilmington, DE, USA, https://www.python.org).

### Modeling and Feature Selection

The random forest algorithm, which belongs to the category of machine learning methods and captures non-line relationships between dependent and independent variables with high flexibility and sufficient accuracy, has been successfully applied to various fields such as the estimation of the genetic effects (23), clinical deterioration prediction (24), association estimation (25), clinical outcome prediction (26), and others (27–30). In this study, we used the random forest algorithm to predict the risk of sepsis in ICU patients by analyzing laboratory/clinic data as follows: (i) lipids, (ii) liver function, (iii) hemagglutination, (iv) blood cells, (v) renal function, and (vi) electrolyte. The essential idea of the random forest algorithm is to build multiple decision trees to reduce the correlation between trees using bootstrap aggregating or bagging, which can avoid the over-fitting problem. The random forest algorithm was written in the Python scripting language (version 3.6.5, Python Software Foundation, Wilmington, DE, USA, https://www.python.org).

Generally, models with more features will achieve higher accuracy than those with fewer features. However, in clinical practice, having more features cannot always improve the performance of the model because of irrelevant or redundant features, which may mislead the models. To recognize the key features and the optimal combination of features, we performed a random forest algorithm on different subsets of the training set. In this study, we identified 55 features, which were potential candidates for sepsis prediction. Because the number of possible feature combinations was large (2^55^), as shown in **Figure 2**, we used the Gini importance to rank the importance of all potential features (31, 32). Specifically, a high Gini importance value was a high priority for incorporation into the model. On the basis of the Gini importance value of each feature, we performed the random forest algorithm on the various feature subsets.

### Validation

In this study, we used a 5-fold cross-validation to assess the prediction performance of the model because it was the most commonly used method for machine learning-based medical problem exploration (33–37). Specifically, the available training set was divided into five roughly equal-sized subsets: the training set and the validation (or internal validation) set. Four of them were applied to fit the random forest model, and the remaining one was used to estimate the accuracy of the model.

We measured the performance of the model by applying several different indices, namely (i) AUC, (ii) accuracy, (iii) precision, (iv) recall, and (v) F1-Score, which were defined as follows:


$$\begin{array}{l} \;Accuracy=\;\frac{TN+TP}{TN+FP+FN+TP}\\ \;Precision=\;\frac{TP}{FP+TP}\\ \;Recall=\;\frac{TP}{FN+TP}\\ \;F1-Score=\;\frac{2\times Precision\times Recall}{Precision+Recall} \end{array}$$

Here, *TP, FP, TN*, and *FN* are the number of positive samples classified as positive (true positives), the number of negative samples classified as positive (false positives), the number of positive samples classified as negative (true negatives), and the number of negative samples classified as negative (false negatives). Briefly, we used five prediction performance indices, 5-fold cross-validation for internal validation, and the testing set for external validation to estimate the performance of the model.

## Results

### Patient Characteristics

**Figure 1:**
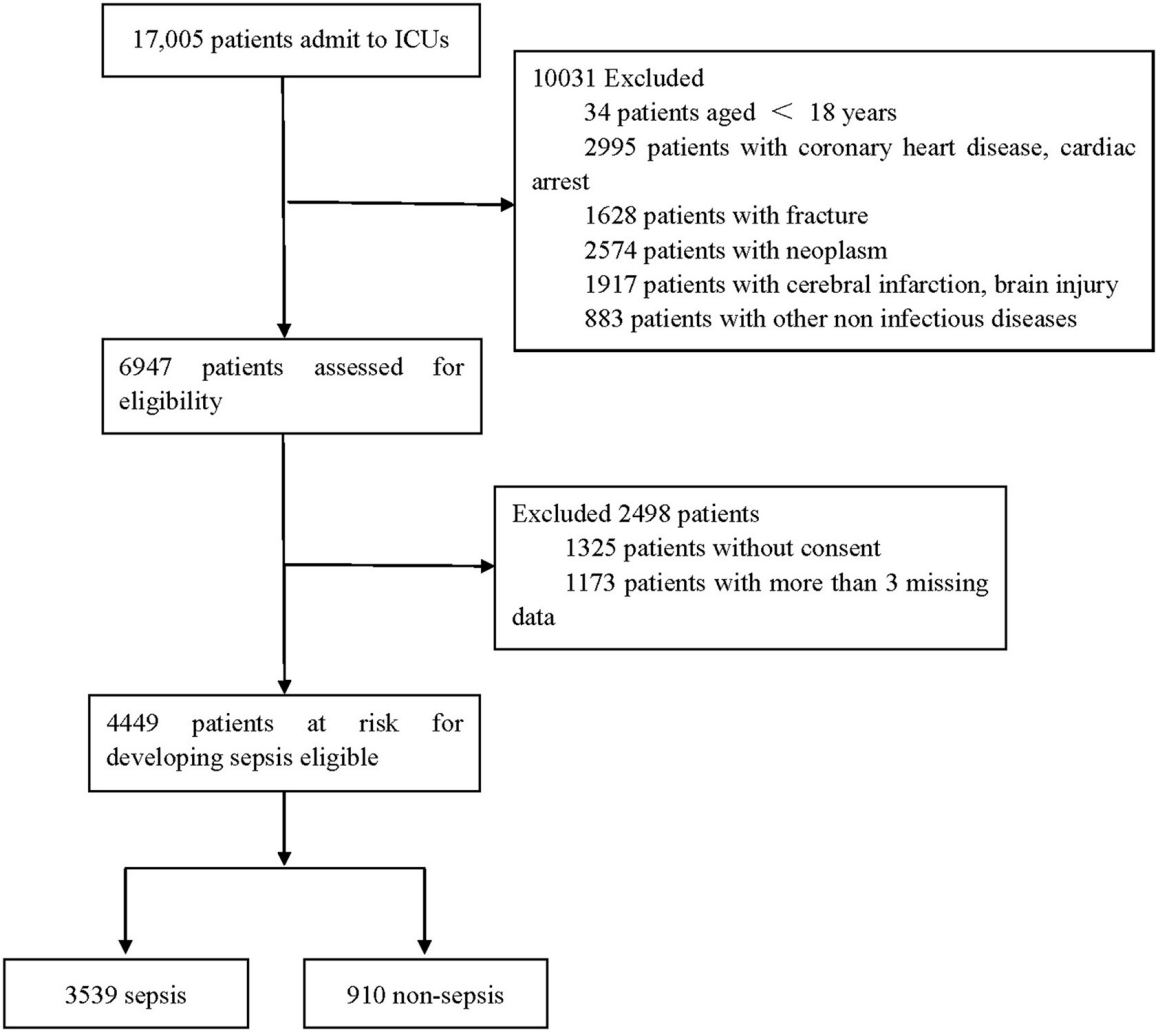
Flow chart depicting number of patients who were included in analysis after exclusion criteria. The total included encounters were divided into those with and without sepsis.

Our database consisted of 17,005 patients admitted to the ICU. After a series of exclusions, 4,449 adult patients were included in this study, and 3,539 patients developed sepsis. The process of cohort selection is shown in Figure 1. A total of 55 variables, including age, sex, red blood cell count, total cholesterol, D-dimer, and other clinical or laboratory parameters related to infection and sepsis, were collected for each patient. The baseline characteristics of the included patients are shown in Supplementary Table 1. We then randomly divided the patients into the training and testing sets. Supplementary Table 2 shows the basic information compared between the two sets.

### Variables of Importance

**Figure 2:**
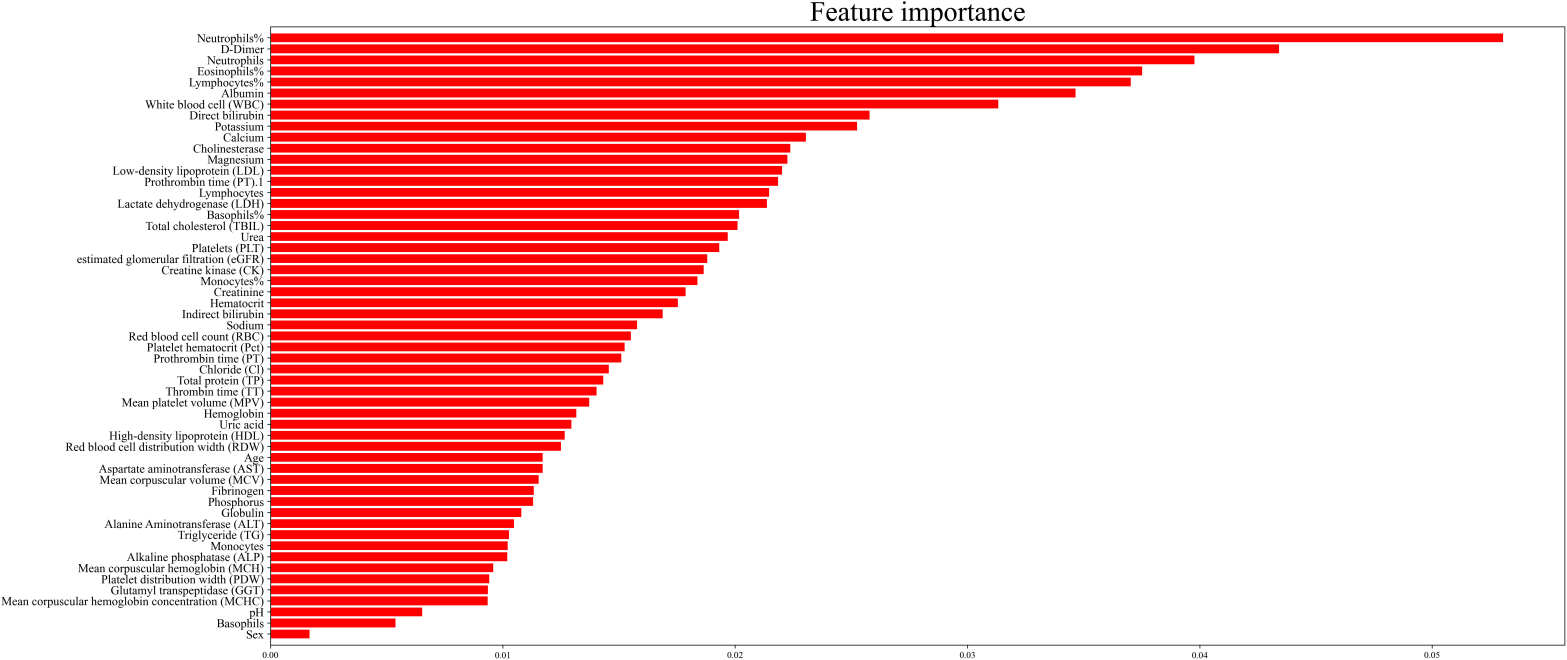
Importance of the 20 variables included in the predictive model for sepsis events.

**Figure 3:**
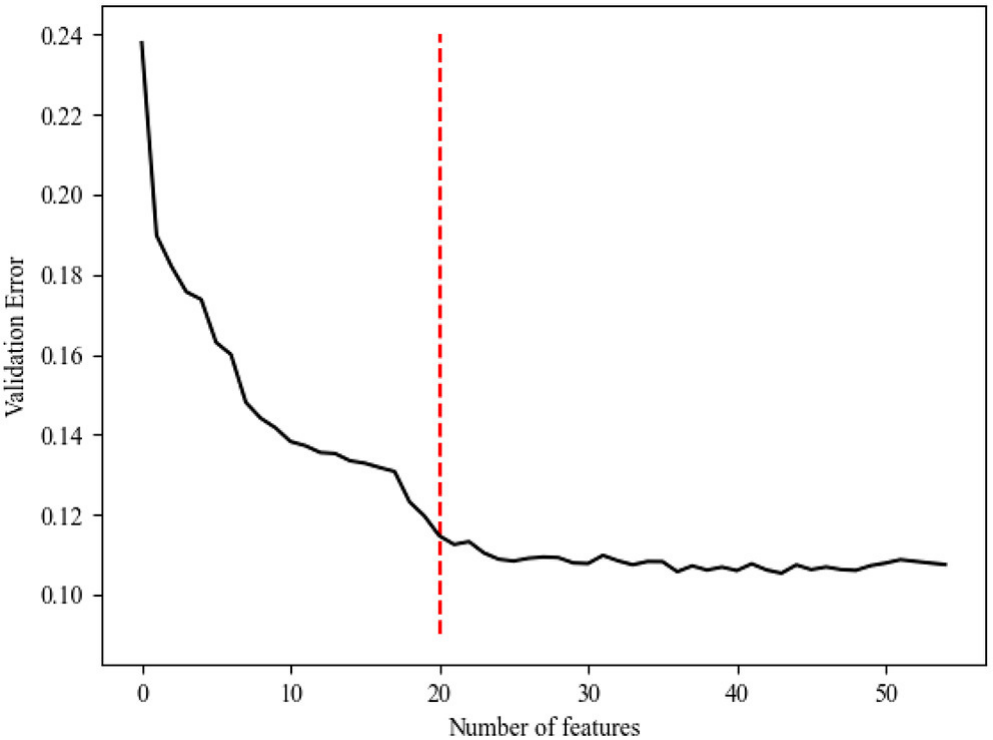
The relationship between the cross-validation error and the number of variables.

Generally, the error of the model decreased with an increase in variable selection. However, increasing the number of variables was not conducive to clinical practice. In order to identify the prominent features, we used the random forest method to select variables by using various feature subsets. Therefore, the relative importance of each feature based on the fact that the features built on the tree top contributed more to the prediction of sepsis in high-risk patients is shown in Figure 2. It can be observed in Figure 3 that the error value remained at a similar degree when the number of features reached 20. Therefore, we utilized a combination of 20 selected features to predict sepsis in ICU patients (shown in Supplementary Table 3): neutrophils%, D-dimer, neutrophils, eosinophils, lymphocytes, albumin, white blood cells (WBCs), direct bilirubin, potassium, calcium, cholinesterase, magnesium, low-density lipoprotein (LDL), prothrombin time (PT), lymphocytes, lactate dehydrogenase (LDH), basophils%, total cholesterol (TBIL), urea, and platelets (PLT).

Next, we performed a random forest classification with the same parameters (to make the comparison possible and remove the effect of the parameters) with different subsets of features to calculate the changes in AUC values. The AUC loss value changed when we set the number of features to different values (Supplementary Table 3).

### Classification Results

**Figure 4:**
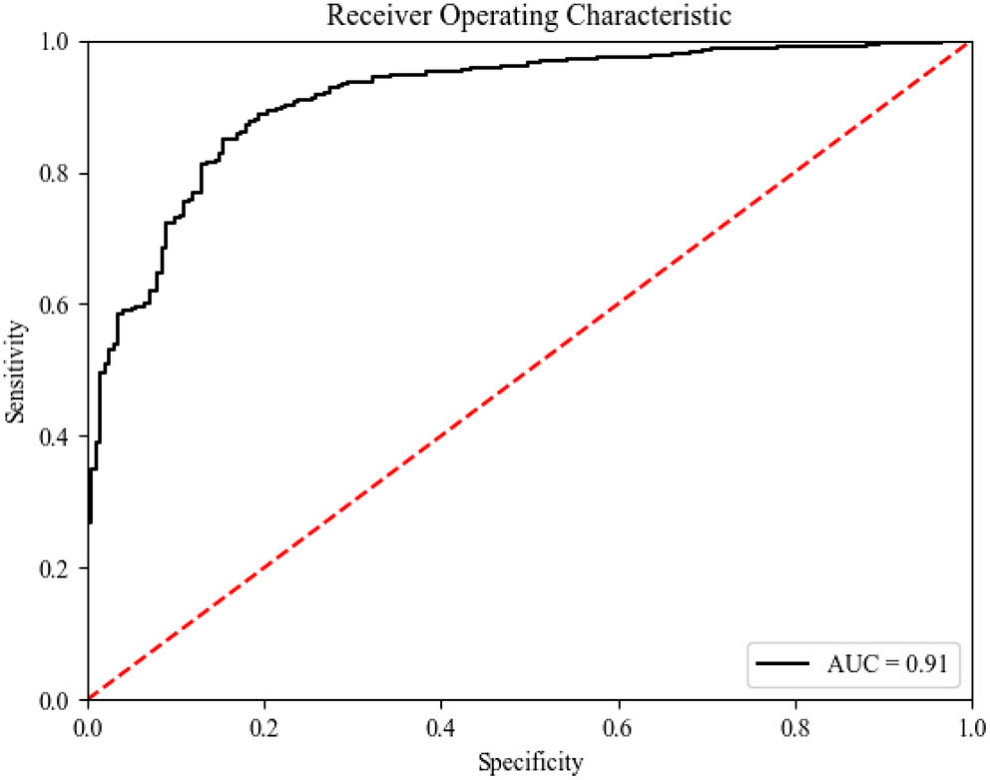
ROC curve (of the testing set) for predicting Sepsis events using the predictive model. ROC receiver operating characteristic.

As shown in Table 1 and Figure 4, on average, the random forest algorithm achieved an AUC of 0.88 (±0.04), accuracy of 0.88 (±0.03), precision of 0.90 (±0.03), recall of 0.96 (±0.01), and recall of 0.93 (±0.02) in the internal validation. For the external validation, the model gave an AUC of 0.91, accuracy of 0.87, precision of 0.89, recall of 0.95, and recall of 0.92.

**Table 1:** Internal and external validation results of the prediction model.

	**AUC**	**Accuracy**	**Precision**
Internal validation/validation set (95% CI)	0.88 (±0.04)	0.88 (±0.03)	0.90 (±0.03)
External validation/testing set	0.91	0.87	0.89
	**Recall**	**F1-Score**	
Internal validation/validation set (95% CI)	0.96 (±0.01)	0.93 (±0.02)	
External validation/testing set	0.95	0.92	

*CI, confidence intervals; AUC, indicates area under the receiver-operating curves*.

## Discussion

Early identification and treatment of sepsis is a highly complex and multifaceted challenge (38). It requires highly skilled and well-trained human experts (39). However, with the continuous emergence of AI applications in the medical field, some of these decisions will soon be replaced by machines called “intelligence” to improve clinical practice and patient outcomes (40). Most of what we call “artificial intelligence” is machine learning, which means learning from data and using this knowledge to acquire new knowledge or skills.

This study used a supervised learning method (a machine learning method) to build a predictive model, which included 20 predictors of sepsis events predicted by the random forest method. The AUC of this newly developed model was 0.91, demonstrating good discriminative power. These prediction results suggest that the ensemble model with 20 key features is feasible and practical.

To our knowledge, most previous studies have developed models to predict the prognosis of sepsis. However, only few researchers have paid attention to the differences in the incidence of sepsis after infection, although it is important for clinical preventive intervention. Thomas et al. developed machine learning models for the early identification of sepsis risk (41); however, they did not obtain precise biomarkers that could be applied to clinicians. All calculations are trivial for a computer, which may limit generalization of the results to other hospitals and hospital systems. Other artificial intelligence systems such as random forest models may be a valuable tool to predict sepsis (8).

The variables in our model were mainly blood cells, lipids, liver function, hemagglutination, renal function, electrolyte, enzyme, and others. Interestingly, blood-related variables accounted for a large part of our model; the first five variables in Figure 2 are related to the blood system. Neutrophils were an ideal choice for eliminating pathogenic bacteria because they store a large number of proteolytic enzymes that can rapidly produce reactive oxygen species to degrade internal pathogens. Hence, patients with sepsis often have neutrophil infiltration, and the degree of infiltration is related to tissue damage (42, 43). Other blood cells, including eosinophils, basophils, lymphocytes, and WBCs, are also associated with the body's defense against infection. For example, some studies have speculated that individuals with basophilic granulocytopenia have a weak resistance to infection and thus are more likely to develop sepsis (44). In addition, studies have shown that eosinophilia was a moderate marker for distinguishing SIRS from infection in critically ill patients newly admitted to the hospital, which suggested that eosinophilia may be a useful clinical tool for the prediction of sepsis (45). In addition, lymphocyte apoptosis has been recognized as an important step in the pathogenesis of experimental sepsis by inducing a state of “immune paralysis” that renders the host vulnerable to invading pathogens (46).

In the past decade, there has been a growing awareness about the role of the coagulation and fibrinolysis systems in the development of inflammation. Patients with sepsis may have common host reactions, such as coagulation, inflammation, and endothelial injury. Abnormal inflammatory and coagulation biomarkers were found to be associated with disease severity and mortality in patients with severe sepsis (47). Platelets are the main effector cells involved in blood coagulation and can promote the development of excessive inflammation, DIC, and microthrombosis (48). PT can reflect the coagulation function of the body, and D-dimer levels increase under hypercoagulable state (49). Therefore, changes in these substances may predict the occurrence of sepsis.

Sepsis is often associated with multiple organ dysfunction such as that involving the heart, liver, and kidney (50). Therefore, some indicators reflecting organ function may be used to predict the occurrence of sepsis. Albumin which is the most important protein in human plasma, maintains nutrition and osmotic pressure. When liver synthesis is dysfunctional, its level usually decreases. Lactate dehydrogenase and urea are associated with cardiac and renal function, respectively. Patel et al. revealed an association between serum bilirubin levels and mortality during sepsis, suggesting that serum bilirubin may be a potential predictor of sepsis occurrence and death (51).

Previous studies have shown that lipids are also involved in the occurrence and development of sepsis. Yamano et al. found that low total cholesterol and high total bilirubin levels are associated with prognosis in patients with prolonged sepsis (52). Hofer et al. found that pharmacologic inhibition of cholinesterase improves survival in experimental sepsis, probably by activating the cholinergic anti-inflammatory pathway (53). The results of Feng's study suggest that a decrease in LDL-C levels is significantly associated with an increased risk of sepsis in infected patients, although the association was due to the presence of complications (54).

Although the association between electrolytes other than calcium and sepsis appears to be poorly studied, this study found that the decrease of potassium and magnesium is closely related to the occurrence of sepsis. We know that the critical illness itself is associated with a decrease in serum total calcium and free calcium levels, which is related to the severity of underlying diseases as measured by the APACHE II score. In addition, studies have shown that total and ionized hypocalcemia is more significantly associated with increased severity of infection, which suggested the role of calcium in predicting the risk of sepsis in patients with infection (55). Regarding magnesium and potassium, a study pointed out that ATP-MgCl_2_ may be beneficial in sepsis (56). An increasing amount of evidence has suggested that potassium channels are involved in cardiovascular dysfunction in sepsis after systemic inflammation, cardiovascular dysfunction, and organ damage, and that potassium channels may affect the emergence of sepsis after infection (57). In conclusion, we believe that because sepsis is not a simple disease that can be predicted by a single marker, the biomarkers included in our model can be combined to predict the risk of sepsis in infected patients.

Our study has several limitations. First, this was a retrospective study, which had its own shortcomings, such as information bias. Second, the prediction model may have lacked generality because the 55 variables are still too few, and many other variables were omitted due to the loss of too many values. Generally, the more the variables included, the higher the prediction accuracy. Therefore, we hope to include more patients and variables in future prospective studies.

A model with 20 key features was successfully established to predict sepsis events in Chinese patients. This model has excellent ability to predict sepsis events in Chinese patients.

## Data Availability Statement

The original contributions presented in the study are included in the article/Supplementary Material, further inquiries can be directed to the corresponding author/s.

## Author Contributions

DW, YS, and JL participated in the research design and coordination and helped to draft the manuscript. XDi and XZ contributed the acquisition of data. JL, SL, BH, HW, XDu, and TS performed the data analysis. All authors contributed to the article and approved the submitted version.

## Funding

This study was supported by grants from the United Fund of National Natural Science Foundation of China (U2004110); Leading Talents Fund in Science and Technology Innovation in Henan Province (194200510017); Science and Technology people-benefit project of Zhengzhou (2019KJHM0001). The special fund for young and middle-aged medical research of China International Medical Exchange Foundation (Z-2018-35); The integrated thinking research foundation of the China foundation for International Medical Exchange (Z-2016-23-2001); The study of mechanism of Gabexate Mesilate in the treatment of sepsis and septic shock (2019-hx-45).

## Conflict of Interest

The authors declare that the research was conducted in the absence of any commercial or financial relationships that could be construed as a potential conflict of interest.

## Publisher's Note

All claims expressed in this article are solely those of the authors and do not necessarily represent those of their affiliated organizations, or those of the publisher, the editors and the reviewers. Any product that may be evaluated in this article, or claim that may be made by its manufacturer, is not guaranteed or endorsed by the publisher.

## Abbreviations

ICU: Intensive care unit
LDL: Low-density lipoprotein
PT: Prothrombin time
ROC: Receiver operating characteristic
WBC: White blood cells.
