# Supplementary material for: A Machine Learning Model for Accurate Prediction of Sepsis in ICU Patients
Source: Front Public Health. 2021 Oct 15;9:754348. doi: 10.3389/fpubh.2021.754348 (PMC8553999; doi:10.3389/fpubh.2021.754348)
Supplement: Supplementary file 1 [file Table_1.DOCX]

**Supplemental Table 1. Demographic, Clinical, and Angiographic Data in Sepsis and Non-sepsis Patients of the Training Data Set**

| **Variable** | **Non-sepsis (n=709)** | **Sepsis (n=2850)** | **P value** |
| --- | --- | --- | --- |
| Sex (male) | 418 (59.0%) | 1735 (60.9%) | 0.349 |
| age | 58.3±14.2 | 57.3±17.1 | 0.118 |
| Prothrombin activity | 99.5±17.7 | 86.7±25.9 | 0.000^*^ |
| Alanine Aminotransferase | 18.0 [12.0,29.0] | 23.0 [13.6,47.0] | 0.000^*^ |
| Chloride | 101±5.2 | 101±8.6 | 0.004^*^ |
| Total protein | 67.3±7.3 | 62.8±10.7 | 0.000^*^ |
| Glutamyl transpeptidase | 26.0 [18.0,48.0] | 33.0 [18.0,71.7] | 0.000^*^ |
| White blood cell | 6.3 [4.8,8.7] | 10.6 [7.1,15.4] | 0.000^*^ |
| Platelet distribution width | 16.7±0.7 | 17.0±0.8 | 0.000^*^ |
| Thrombin time | 14.3±3.5 | 15.6±7.5 | 0.000^*^ |
| Aspartate aminotransferase | 21.0 [16.0,30.0] | 30.0 [19.0,64.8] | 0.000^*^ |
| Alkaline phosphatase | 78.0 [63.0,98.6] | 79.9 [61.0,110] | 0.147 |
| Lymphocytes% | 23.1 [14.8,32.1] | 8.50 [4.8,16.3] | 0.000^*^ |
| Neutrophils% | 65.2±15.3 | 79.5±16.2 | 0.000^*^ |
| High-density lipoprotein | 1.2±0.4 | 1.0±0.5 | 0.000^*^ |
| Monocytes | 0.5 [0.4,0.7] | 0.6 [0.3,0.9] | 0.000^*^ |
| MCHC | 332±9.5 | 332±13.1 | 0.926 |
| Direct bilirubin | 3.7 [2.7,5.2] | 5.9 [3.8,11.2] | 0.000^*^ |
| Urea | 4.9 [3.9,6.0] | 6.5 [4.6,10.3] | 0.000^*^ |
| Triglyceride | 1.1 [0.8,1.7] | 1.1 [0.8,1.6] | 0.391 |
| Low-density lipoprotein | 2.7 [2.1,3.4] | 2.1 [1.4,2.7] | 0.000^*^ |
| Globulin | 28.1±6.2 | 29.1±7.6 | 0.000^*^ |
| Eosinophils% | 1.4 [0.4,2.9] | 0.2 [0,0.8] | 0.000^*^ |
| Creatinine | 65.0 [56.0,77.0] | 68.0 [52.0,104] | 0.000^*^ |
| Albumin | 39.2±5.9 | 33.6±7.4 | 0.000^*^ |
| Prothrombin time | 11.2±1.8 | 13.8±10.5 | 0.000^*^ |
| Basophils | 0.02 [0.01,0.04] | 0.01[0,0.03] | 0.000^*^ |
| Magnesium | 1.0±0.1 | 0.9±0.2 | 0.000^*^ |
| D-Dimer | 0.3 [0.1,0.6] | 0.9 [0.4,2.8] | 0.000^*^ |
| Hemoglobin | 124±20.5 | 114±30.0 | 0.000^*^ |
| Lymphocytes | 1.4 [1.0,1.8] | 0.9 [0.5,1.4] | 0.000^*^ |
| Cholinesterase | 6.6±2.15 | 5.3±2.4 | 0.000^*^ |
| Fibrinogen | 3.5±0.7 | 3.5±1. 3 | 0.738 |
| Uric acid | 272±95.5 | 312±175 | 0.000^*^ |
| Hematocrit | 0.4 [0.3,0.4] | 0.4 [0.3,0.4] | 0.000^*^ |
| pH | 5.9±0.7 | 5.9±0.7 | 0.662 |
| Potassium | 4.2±0.6 | 4.0±0.9 | 0.000^*^ |
| Indirect bilirubin | 4.0 [2.8,6.2] | 6.1[3.7,10.2] | 0.000^*^ |
| Creatine kinase | 60.0 [41.0,82.0] | 81.0 [53.0,257] | 0.000^*^ |
| Lactate dehydrogenase | 215 [177,265] | 265 [220,426] | 0.000^*^ |
| Neutrophils | 4.0 [2.7,6.4] | 8.8 [5.2,13.5] | 0.000^*^ |
| Platelets | 229±90.5 | 184±114 | 0.000^*^ |
| Mean platelet volume | 8.5±1.3 | 8.9±1.4 | 0.000^*^ |
| Total cholesterol | 8.0 [5.6,11.5] | 12.7 [8.0,22.3] | 0.000^*^ |
| Estimated glomerular filtration | 95.4±18.0 | 84.3±37.3 | 0.000^*^ |
| Sodium | 141±5.0 | 139±7.6 | 0.000^*^ |
| RDW | 14.8±2.2 | 15.2±2.8 | 0.000^*^ |
| Red blood cell count | 4.1±0.7 | 3.7±1.0 | 0.000^*^ |
| Basophils% | 0.4 [0.3,0.6] | 0.1 [0,0.3] | 0.000^*^ |
| Platelet hematocrit | 0.19±0.07 | 0.16±0.09 | 0.000^*^ |
| MCH | 30.6±2.1 | 30.7±2.8 | 0.178 |
| Phosphorus | 1.2±0.3 | 1.1±0.6 | 0.091 |
| Monocytes% | 8.2 [6.2,10.4] | 5.7 [3.5,8.1] | 0.000^*^ |
| MCV | 91.8±6.1 | 92.2±7.4 | 0.151 |
| Calcium | 2.2±0.1 | 2.1±0.2 | 0.000^*^ |

The binary variables are described as counts and percentages and were evaluated by the Chi-squared test or Fisher’s exact test. Continuous variables of each group are presented as the mean ± SEM / Median [interquartile range]. Student’s t-test was used to compare the normally distributed continuous variables; Mann–Whitney U test is used to compare non-normally distributed continuous variables.

MCHC, Mean corpuscular hemoglobin concentration; RDW, Red blood cell distribution width; MCH, Mean corpuscular hemoglobin; MCV, Mean corpuscular volume.

*P < 0.05, Sepsis compared with non-Sepsis.

**Supplemental Table 2. Baseline characteristics and clinical/laboratory parameters in the training and testing cohorts**

| **Variable** | **Training cohorts (n=3559)** | **Testing cohorts (n=890)** | **P value** |
| --- | --- | --- | --- |
| Neutrophils% | 76.6±17.0 | 75.7±17.2 | 0.161 |
| D-Dimer | 0.7 [0.3,2.4] | 0.7 [0.3,2.5] | 0.924 |
| Neutrophils | 7.7 [4.1,12.4] | 7.4 [4.0,12.1] | 0.305 |
| Eosinophils% | 0.3 [0,1.2] | 0.3 [0,1.3] | 0.101 |
| Lymphocytes% | 10.4 [5.3,20.7] | 10.9 [5.5,22.8] | 0.127 |
| Albumin | 34.7±7.5 | 34.8±7.6 | 0.701 |
| White blood cell | 9.5 [6.1,14.4] | 9.5 [6.0,14.1] | 0.391 |
| Direct bilirubin | 5.3 [3.4,9.5] | 5.0 [3.3,9.0] | 0.077 |
| Potassium | 4.1±0.8 | 4.1±0.8 | 0.419 |
| Calcium | 2.2±0.2 | 2.2±0.2 | 0.822 |
| Cholinesterase | 5.5±2.4 | 5.6±2.4 | 0.662 |
| Magnesium | 0.9±0.2 | 0.9±0.2 | 0.658 |
| Low-density lipoprotein | 2.2±1.1 | 2.2±1.2 | 0.645 |
| Prothrombin time | 13.3±9.5 | 13.3±7.9 | 0.962 |
| Lymphocytes | 0.97 [0.60,1.53] | 1.02 [0.64,1.54] | 0.194 |
| Lactate dehydrogenase | 263 [204,395] | 263 [200,392] | 0.378 |
| Basophils% | 0.2 [0.1,0.4] | 0.2 [0.1,0.4] | 0.298 |
| Total cholesterol | 11.4 [7.3,19.5] | 10.7 [6.9,18.9] | 0.044^*^ |
| Urea | 6.0 [4.3,9.1] | 6.0 [4.3,9.6] | 0.627 |
| Platelets | 193±111 | 199±111 | 0.170 |

Continuous variables of each group are presented as the mean ± SEM / Median [interquartile range]. Student’s t-test was used to compare the normally distributed continuous variables; Mann–Whitney U test is used to compare non-normally distributed continuous variables. *P < 0.05.

**Supplemental Table 3. The relationship between the AUC loss and the number of features.**

| **Feature Name** | **AUC Loss** |
| --- | --- |
| Neutrophils% | 0.238 |
| D-Dimer | 0.190 |
| Neutrophils | 0.182 |
| Eosinophils% | 0.176 |
| Lymphocytes% | 0.174 |
| Albumin | 0.163 |
| White blood cell | 0.160 |
| Direct bilirubin | 0.148 |
| Potassium | 0.144 |
| Calcium | 0.142 |
| Cholinesterase | 0.138 |
| Magnesium | 0.137 |
| Low-density lipoprotein | 0.136 |
| Prothrombin time | 0.135 |
| Lymphocytes | 0.134 |
| Lactate dehydrogenase | 0.133 |
| Basophils% | 0.132 |
| Total cholesterol | 0.131 |
| Urea | 0.123 |
| Platelets | 0.120 |

AUC, indicates area under the receiver-operating curves.
